# The impact of CDIO implementation on retrofitting CNC based universal turn drill in vocational education

**DOI:** 10.1016/j.mex.2025.103547

**Published:** 2025-08-05

**Authors:** Widodo, Suhardjono, M. Khoirul Effendi

**Affiliations:** a Mechanical Engineering Department, Faculty of Industrial Technology and Systems Engineering, Institut Teknologi Sepuluh Nopember, Surabaya, 60111, Indonesia; b Mechanical Engineering Department, Politeknik Negeri Batam, Batam, Indonesia

**Keywords:** CDIO, CNC, Retrofit, Vocational

## Abstract

The CDIO approach had been implemented in the CNC lathe retrofit manufacturing process to support learning in vocational schools, such as SMK and polytechnics. This research aims to evaluate the improvement of technology-based student competencies that are relevant to the needs of industry 4.0. The stages in this method are divided into three, namely the initial identification of the problem or project, designing the model and electrical sub-components and analyzing the improvement of process evaluation results. The implementation actively involves the collaboration of 4–6 students from various cross majors by appointing one student as a leader who is supervised by a lecturer as a project manager and an instructor as a project facilitator. Validation of the project takes place upon completion of the project activities, with student groups submitting a report detailing the project implementation design, logbook, and final deliverables. This documentation is comprehensively reviewed by the project manager, faculty team, and an interdisciplinary expert committee to assess the validation results of the activity, which will be integrated into the relevant course assessment. This evaluation and validation review relates to a 13-point institutionally developed instrument that is linear to the discipline-specific graduate outcomes. The results of the analysis show that the majority of respondents from students and lecturers agree that the CDIO approach strengthens the understanding of technical concepts and practical skills through structured learning stages, from designing to operating prototype products. In addition, it can also integrate through aspects of collaboration, problem solving, and innovation that support the needs of modern industry.

• The implementation of CDIO (Conceive, Design, Implement, Operate) is essential in vocational faculty, vocational high schools (SMK), and polytechnics, particularly for CNC-based universal turn drill retrofit manufacturing projects, as this methodology promotes practice-oriented learning, industry relevance, and aligns with a 12-standard framework
• Through a Likert scale-based data collection method, this study measured the level of understanding, technical skills, and user satisfaction with the concept of CNC retrofitting.
• The success of this method is measured through several parameters, including increased student competence, active involvement in the project, and the final product produced with an average approval percentage score above 70 %.

## Graphical abstract

**Figure:**
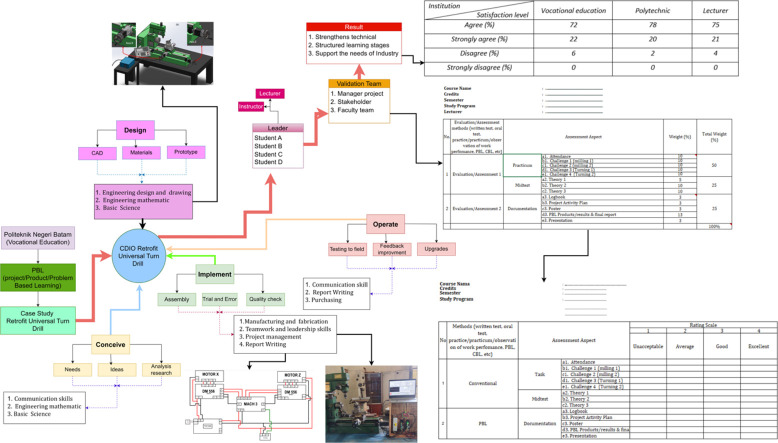
The global concept of the applied CDIO retrofit CNC turn drill machine.

**Standard and Syllabus of CDIO**

**Table 1:** Standard CDIO.

No	CDIO Standards	Standard Description
1	CDIO in Context	This standard highlights the significance of the life cycle development context of products, processes, and systems in engineering education, specifically through the stages of Conceiving, Designing, Implementing, and Operating.
2	Outcomes of the CDIO Syllabus	This standard delineates explicit learning objectives for individual and relational competencies alongside competencies in the development of products, processes and systems aligned with program objectives.
3	Comprehensive Curriculum	This standard highlights the necessity of an integrated curriculum that interconnects various disciplines to facilitate a more comprehensive learning experience for students.
4	Fundamental of technology	This standard provides an introductory overview of engineering principles and practices for new students, ensuring a foundational understanding prior to engaging in more advanced studies.
5	Design build experiences	The standard highlights the significance of incorporating design and implementation experiences within the curriculum, enabling students to apply their knowledge to real-world projects.
6	CDIO Workshop	This standard emphasizes the provision of suitable learning environments to facilitate active and collaborative learning among students.
7	Educational Experiences	This standard promotes integrated learning experiences, enabling students to acquire knowledge from diverse contexts and real-life scenarios.
8	Activity based learning	This standard promotes the adoption of innovative teaching and learning methods to augment student involvement and education outcomes.
9	Improvement of department CDIO competencies	This standard emphasizes the development of faculty competencies to ensure they possess the requisite skills for effective teaching within the CDIO framework.
10	Improvement of department instructional proficiencies	focused on enhancing faculty teaching skills to present material in an engaging and effective manner
11	CDIO competency evaluation	This standard highlights the necessity of thorough learning assessment to effectively measure the attainment of student learning outcomes.
12	Evaluation of the CDIO scheme	The final standard focuses on evaluating the program as a whole to ensure that all aspects of the CDIO program are working in accordance with established objectives and provide feedback for continuous improvement.

**Table 2:** CDIO Syllabus v2.0.

Item	Description
Disciplinary expertice	This category includes the basic knowledge required in engineering, Mathematics: Understanding of mathematical concepts and statistics. Natural Sciences: Knowledge of physics, chemistry, and biology relevant to engineering Technology: Understanding of technological systems used in industry
Skill in the development of product, processes, systems and services	This category includes the practical skills required to design and interpretation products, proceures, systems and services, i.e. Design: The ability to design technical solutions that meet specific needs. Implementation: Skills in implementing a design into a real product or system. Operation: Being able to operate and maintain a system or product after implementation.
Individual and social competencies	This category focuses on developing individual and relational competencies that are important in the work environment, namely Communication Skills: The capacity for effective communication with various parties.
Contextual understanding	This category relates to understanding the social, economic and environmental context in which techniques are applied, i.e. Social Awareness: Understanding the social impact of Engineering technologies and decisions Professional Ethics: Recognizing ethical responsibilities as an engineer. Sustainability: Knowledge of sustainable practices and their impact on the environment.

**Fig. 1:**
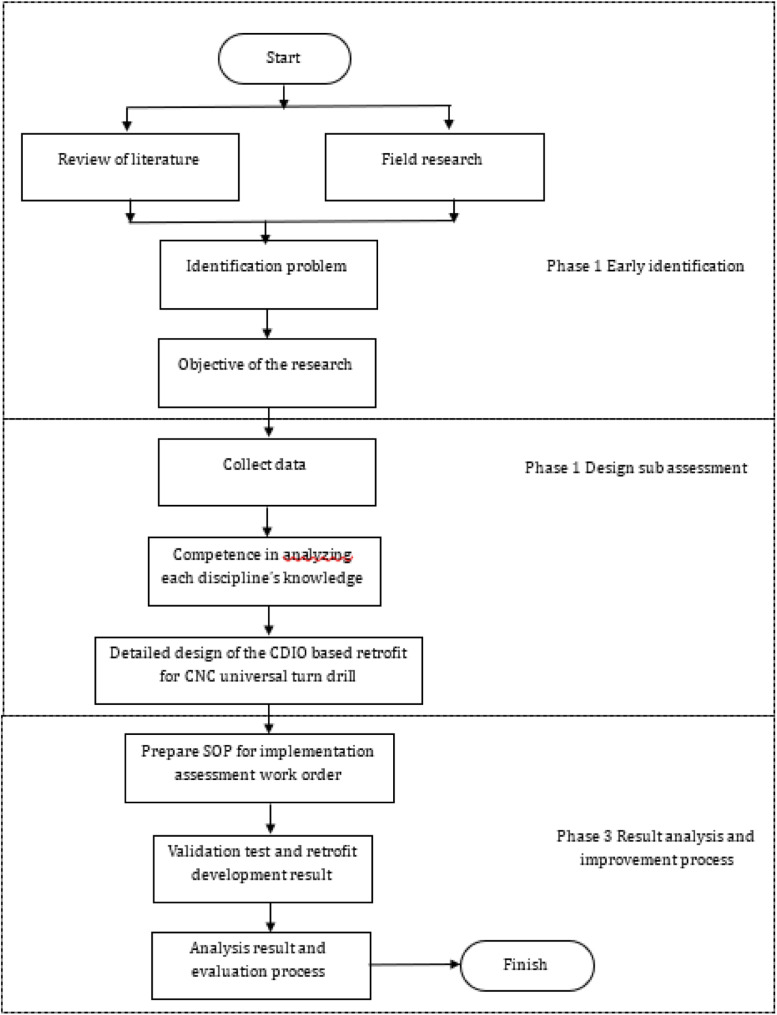
Flow diagram for resolving the CNC universal turn drill retrofit (Bachtiar & others, 2018).

**Fig. 2:**
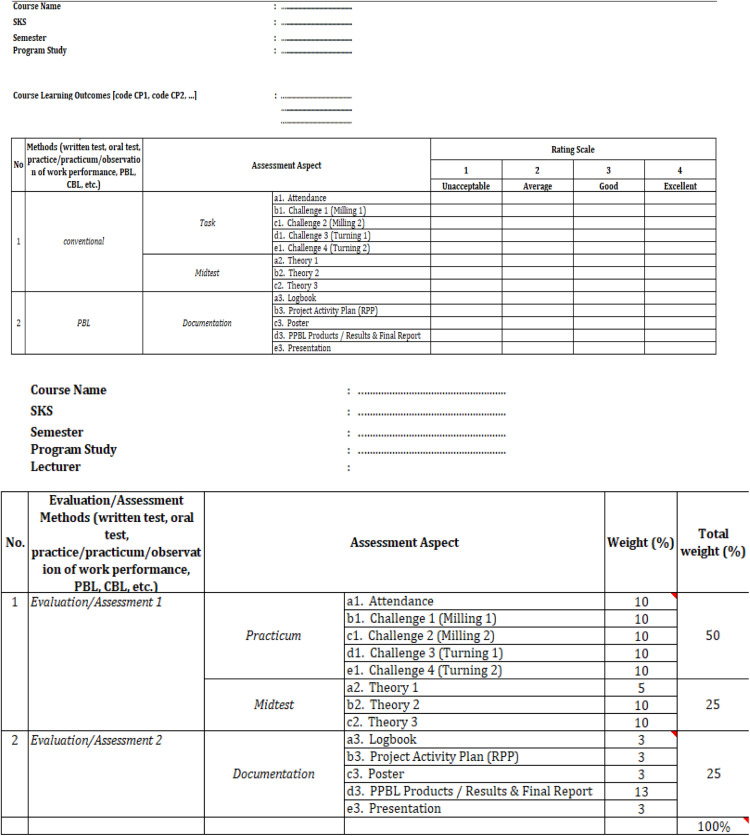
Final assessment rubric for CNC course [3].

**Specifications table**

**Table 3:** 

**Subject area:**	Engineering
**More specific subject area:**	The CDIO concept for vocational education implementation
**Name of your method:**	Conceive Development Implement Operate (CDIO)
**Name and reference of original method:**	Bachtiar, Y., & others. (2018). Penerapan konsep CDIO pada praktikum pemrograman komputer sebagai media pembelajaran kreatif dan aplikatif. Ph.D. dissertation, Universitas Muhammadiyah Surakarta. Baryshev, G., Yudin, I., Biryukov, A., Shukin, B., & Yultyeva, R. (2021). Application of the CDIO standards for cyber-physical education in mechatronics and robotics in a research university on the example of development of digital electronic skills. Procedia Computer Science, 190, 45–50. Elamvazuthi, I., Lee, H. J., Ng, J. C., Song, H. L., Tiong, Y. X., Parimi, A. M., & Swain, A. K. (2015). Implementation of a new engineering approach for undergraduate control system curriculum using a robotic system. Procedia Computer Science, 76, 34–39. Gunnarsson, S. (2017). Automatic control education in a CDIO perspective. IFAC-PapersOnLine, 50, 12,161–12,166. Haavi, T., Tvenge, N., & Martinsen, K. (2018). CDIO design education collaboration using 3D-desktop printers. Procedia CIRP, 70, 325–330. Kulkarni, S., Patil, S., & Pawar, R. (2020). Adoption of the Conceive-Design-Implement-Operate approach to the Third Year Project in a team-based design-build environment. Procedia Computer Science, 172, 559–567. Lai, C.-F., Zhong, H.-X., & Chiu, P.-S. (2021). Investigating the impact of a flipped programming course using the DT-CDIO approach. Computers & Education, 173, 104,287. Lee, C. H., Lee, L., & Kuptasthien, N. (2018). Design thinking for CDIO curriculum development. Proceedings of the 14th International CDIO Conference. Kanazawa, Japan: Kanazawa Institute of Technology, (pp. 88–98). Lingling, G., Guowei, T., Yu, F., Jinghui, L., & Wanping, Z. (2012). Research and practice on CDIO-based application-oriented practical teaching system of computer major. IERI Procedia, 2, 24–29. Power, J., Tanner, D., Ryan, A., & Devitt, B. (2019). Developing CDIO practitioners: a systematic approach to standard 10. Procedia Manufacturing, 38, 680–685. Quist, J., Bhadani, K., Bengtsson, M., Evertsson, M., Malmqvist, J., Enelund, M., & Hoffenson, S. (2017). CDIO based engineering design and optimization course. Proceedings of the 13th International CDIO Conference, Calgary, June 18–22, 2017., (pp. 298–314). Shuhaiber, A., & Aldwairi, M. (2022). The impact of CDIO's dimensions and values on IT Learner's attitude and behavior: A regression model using Partial Least Squares. Heliyon, 8. Song, D. (2018). Comparison of CDIO and Chinese engineering education accreditation for animation specialty of TUST. Procedia computer science, 131, 765–770. Zelmiyanti, R., Irianto, D., Riadi, S., & others. (2024). CDIO Framework: A Solution of The Project-Based Learning Problem in Accounting Study Program. JOURNAL OF APPLIED MANAGERIAL ACCOUNTING, 8, 146–155.
**Resource availability**	–

## Background

Politeknik Negeri Batam (Polibatam) is a vocational education institution in the Riau Islands, Indonesia, which significantly contributes to fostering a knowledge-based environment and developing soft skills in alignment with industry requirements. This institution's educational approach initially emphasized project/problem/product-based learning (PBL), focusing on real or need-based issues, wherein students must devise solutions and create products as learning outcomes (Aristin, Hastuti, Arisanty, Adyatma, & Donna, 2023) (Helmi, et al., 2024). Numerous research addressing PBL indicate that this pedagogical approach enhances vocational students' critical thinking, cooperation, and problem-solving abilities in practical contexts. Furthermore, the importance of Project-Based Learning (PBL) in steering societal advancement towards sustainability can cultivate essential practical skills for the workforce, while also enhancing conceptual and adaptive comprehension required in a dynamic context (Aryan & Shettar, 2023) (Lubis, et al., 2022) (Omelchenko, 2021). Due to the complexity of problems and the wide range of project types from various industries, the courses that are taught in class emphasize the concepts of conceiving, designing, implementing, and operating (CDIO). (Bachtiar & others, 2018) [1]. This teaching method was first introduced by MIT with the intention enhancing the quality of education and learning as well as the ability of students to solve problems in an active and innovative manner. In other words, it provides a structured and skematic learning environment so that it is the student’s individual skill not only theoretical knowledge that is acquired but also practical application [2]. The constraint additionally, CDIO helps increase and minimizes throughout project execution. In light of this, polibatam is one of the few Indonesian institutions that participates in the global CDIO collaboration framework. As a result, in a few years later, this institution is referred to as an active workshop by a few Indonesian vocational schools, specifically focusing on politechnic (Zelmiyanti, Irianto, Riadi, & others, 2024). This institution is made up of eight departments, each of which oversees a few academic programs. These departments consist of mechanical, electrical, information technology, and business management departments. The implementation of CDIO in this institution is based on data from the third semester of the current year. Internal and stakeholder project requests are becoming more frequent as the industrial population grows with increased competence, as shown in Table 4. As a result, this potential must be used for the local development of human resources (HRD), mostly through vocational education, with the industry eventually adopting HRD that meets their needs and competencies. In the line with this policy, the Indonesian government, through the Directorate of vocational education, has also approved and implemented the link match program, which is intended to strengthen the relationship between education and industry through (https://vokasi.kemdikbud.go.id, 2024).

**Table 4:** The number best of educational project in Polibatam.

Department	Semester		
	Even ‘22/23	Odd‘23/24	Even ‘23/24
Mechanical engineering	99	86	290
Electrical engineering	201	319	274
Informatics engineering	199	167	202
Business management	58	80	58
Project total	557	652	824

Source: [3].

**Fig. 3:**
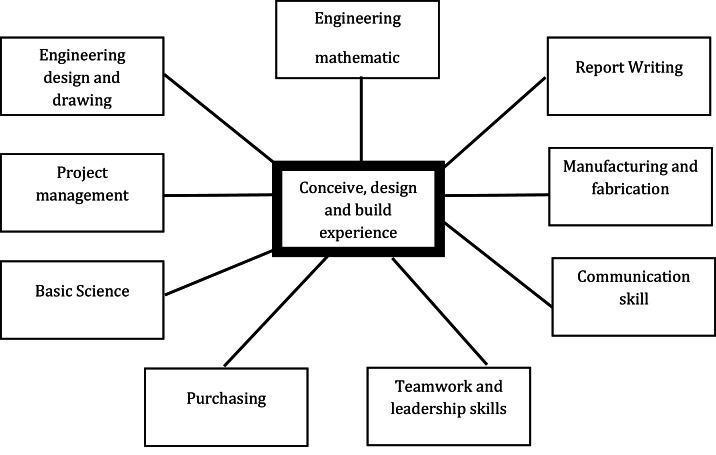
The global concept of the applied CDIO [6].

The concept CDIO as shown in Fig. 3 is a clear and concise manner. In this concept, various knowledge elements, including soft skills and hard skills, are used. This competency is based on character development and strengthen based on problem-based learning (PBL) through the application of understanding concepts from conception, design, implementation, and operation. In addition to this, it also highlights the active participation of both the instructors and the organization in order to facilitate and specifically assess the skills of each and every student participating in the activities. Accordingly, CDIO is extremely important and significant in raising the educational quality implementation that are implemented in Indonesia vocational educational system. Moreover, CDIO is more appropriate and suitable for since it emphasizes PBL-based learning more thoroughly, adhering to the established syllabus and 12 standards [1,4]. According to the framework of international accreditation, this syllabus and standard are essentially linear. In other words, CDIO training at this vocational school will be easier for students to develop their skills, and technical assistance for PBL tasks will be used to help administrators get international accreditation. Hence, the implementation of this CDIO can facilitate the development of the skills that are needed as the demands of the Industry 4.0 grow. The CDIO concept is an innovative approach to education that aims to enhance the quality of technical education and prepare students for the workplace demands. (Hadi, Arifin, Munadi, & others, 2024) (Jalil, Razali, Rahman, Rahim, & Abd Samad, 2024). In addition, CDIO can increase students' engagement in the learning process and help them develop their problem-solving skills more effectively. A few examples of the benefits or drawbacks of CDIO's implementation are summarized below [5]

Improving Student Skills to improve the quality of the work of the students, the link and match concept will be discussed. This is based on the interaction between the business and industry, which are representatives of the vocational school.As a result of this link and match, the government has taken action in a tax insentif program to the active industrial sector, which will benefit education in vocational schools.

Suitability to Industry

One of the main challenges in vocational education is the tension between the knowledge being taught and the demands of industry. By using CDIO, the curriculum may be easily adjusted to the work environment. This helps them come up with more concise and relevant to graduated students, which increases their ability to work.

Needs and Problem-based learning

CDIO encourages the implementation of project-based learning that allows students to acquire knowledge through practical experience. This method not only deepens concept understanding, but also hones problem-solving and collaboration skills among students. This methodology is vital within the framework of Industry 4.0, where digital and collaborative competencies are becoming indispensable.

Student Competency Development

In addition to placing an emphasis on the development of technical abilities, CDIO also places an emphasis on the development of soft skills such as leadership, communication, and teamwork. These are the kinds of talents that are absolutely necessary for achieving success in a dynamic and constantly shifting work environment. As a result, students not only develop expertise in engineering but also acquire the ability to adjust to a wide range of circumstances that may arise in the workplace.

Collaboration with Industrial

The implementation of CDIO fosters enhanced collaboration between educational institutions and industry. This collaboration enhances students' educational experience and ensures that vocational education aligns with current industrial advancements.

The curriculum developed in alignment with the CDIO approach and integrated with the government's flagship program is expected to enhance synergy with industry requirements.

## Conceive design implement operate (CDIO)

### Standard and syllabus of CDIO

To guarantee the efficient application of CDIO vocational education, there are 12 standards that have been developed to provide a guiding sequence and reference points for the necessary groups competency [7]. The standards in CDIO represent critical attributes of a vocational education development program, as illustrated in Table 5. The CDIO standards highlight the importance of curriculum development and enhancement, program fortification, engagement in project/problem/product-based learning (PBL), pedagogical advancement, learning methodologies, assessment evaluation, and the creation of study programs that are responsive to contemporary needs [8]

**Table 5:** Standard CDIO.

No	CDIO Standards	Standard Description
1	CDIO in Context	This standard highlights the significance of the life cycle development context of products, processes, and systems in engineering education, specifically through the stages of Conceiving, Designing, Implementing, and Operating.
2	Outcomes of the CDIO Syllabus	This standard delineates explicit learning objectives for individual and relational competencies alongside competencies in the development of products, processes and systems aligned with program objectives.
3	Comprehensive Curriculum	This standard highlights the necessity of an integrated curriculum that interconnects various disciplines to facilitate a more comprehensive learning experience for students.
4	Fundamental of technology	This standard provides an introductory overview of engineering principles and practices for new students, ensuring a foundational understanding prior to engaging in more advanced studies.
5	Design build experiences	The standard highlights the significance of incorporating design and implementation experiences within the curriculum, enabling students to apply their knowledge to real-world projects.
6	CDIO Workshop	This standard emphasizes the provision of suitable learning environments to facilitate active and collaborative learning among students.
7	Educational Experiences	This standard promotes integrated learning experiences, enabling students to acquire knowledge from diverse contexts and real-life scenarios.
8	Activity based learning	This standard promotes the adoption of innovative teaching and learning methods to augment student involvement and education outcomes.
9	Improvement of department CDIO competencies	This standard emphasizes the development of faculty competencies to ensure they possess the requisite skills for effective teaching within the CDIO framework.
10	Improvement of department instructional proficiencies	focused on enhancing faculty teaching skills to present material in an engaging and effective manner
11	CDIO competency evaluation	This standard highlights the necessity of thorough learning assessment to effectively measure the attainment of student learning outcomes.
12	Evaluation of the CDIO scheme	The final standard focuses on evaluating the program as a whole to ensure that all aspects of the CDIO program are working in accordance with established objectives and provide feedback for continuous improvement.

The syllabus serves as a crucial element within the CDIO framework, delineating the anticipated learning outcomes. The CDIO Syllabus 2.0 delineates four primary categories encompassing essential skills and knowledge required for vocational students, as illustrated in Table 6 [5],[9]

**Table 6:** CDIO Syllabus v2.0.

Item	Description
Disciplinary expertice	This category includes the basic knowledge required in engineering, ✔ Mathematics: Understanding of mathematical concepts and statistics. ✔ Natural Sciences: Knowledge of physics, chemistry, and biology relevant to engineering ✔ Technology: Understanding of technological systems used in industry
Skill in the development of product, processes, systems and services	This category includes the practical skills required to design and interpretation products, proceures, systems and services, i.e. ✔ Design: The ability to design technical solutions that meet specific needs. ✔ Implementation: Skills in implementing a design into a real product or system. ✔ Operation: Being able to operate and maintain a system or product after implementation.
Individual and social competencies	This category focuses on developing individual and relational competencies that are important in the work environment, namely ✔ Communication Skills: The capacity for effective communication with various parties.
Contextual understanding	This category relates to understanding the social, economic and environmental context in which techniques are applied, i.e. ✔ Social Awareness: Understanding the social impact of Engineering technologies and decisions ✔ Professional Ethics: Recognizing ethical responsibilities as an engineer. ✔ Sustainability: Knowledge of sustainable practices and their impact on the environment.

The syllabus and standards in CDIO significantly influence the development of a framework of instruments for each learning activity, facilitating a sequence of processes leading to a final evaluation aligned with the desired competencies.

### Integration of CDIO into vocational education learning

The initial step in implementing the CDIO framework involves identifying or supplying projects, problems, or products derived from internal requests or stakeholder input, focusing on topics that enhance the competencies of each study program or facilitate the integration of cross-disciplinary fields. The Polibatam mechanical engineering department initiates project distribution activities for student teams at the start of the second year, or third semester. At the outset of the first year or semester, students are enriched and strengthened in their understanding of concepts and theories, as well as foundational knowledge in mechanical engineering and related sciences. This process fosters maturity in cognitive patterns and methodologies, enabling the successful completion of PBL activities within a single semester through structured sequences and schemes. This learning concept highlights the importance of multidisciplinary collaboration between basic science and engineering, as illustrated in Fig. 3 [10],[11]. The primary objective is to address the identification of needs in practical field conditions to develop a system or product required by users and industry. CDIO addresses the challenge posed by the variety of activities present in project-based learning (PBL). This method is guided by the review of the complexity and difficulty of activities, adjusting for the competency and education levels of current students. Therefore, collaboration across scientific disciplines and educational generations is essential for the successful completion of each PBL activity within this series of processes, adhering to the following stages:

Conceive

Conceive is one of the steps to understanding the project requirements. The initial phase typically involves the formulation of a project idea, referred to as the creation stage.

Design

The procedure involves generating a design through sketches or drawings that effectively describe the project and delineate the necessary components.

Implement

Testing and validation occur subsequent to the application of project sketches or drawings, representing the process of transforming designs into products.

Operate

This step entails executing projects that are based on prior implementations. Assessments will be conducted to monitor the project's future improvement or progress.

The steps undertaken by Polibatam to integrate the ongoing curriculum into CDIO include curriculum planning, development of active learning, training for lecturers and instructors, evaluation of feedback, and collaboration with business and industry partners. (Zelmiyanti, Irianto, Riadi, & others, 2024)

### Implementation of CDIO for the retrofitting project of universal turn drill

**Fig. 4:**
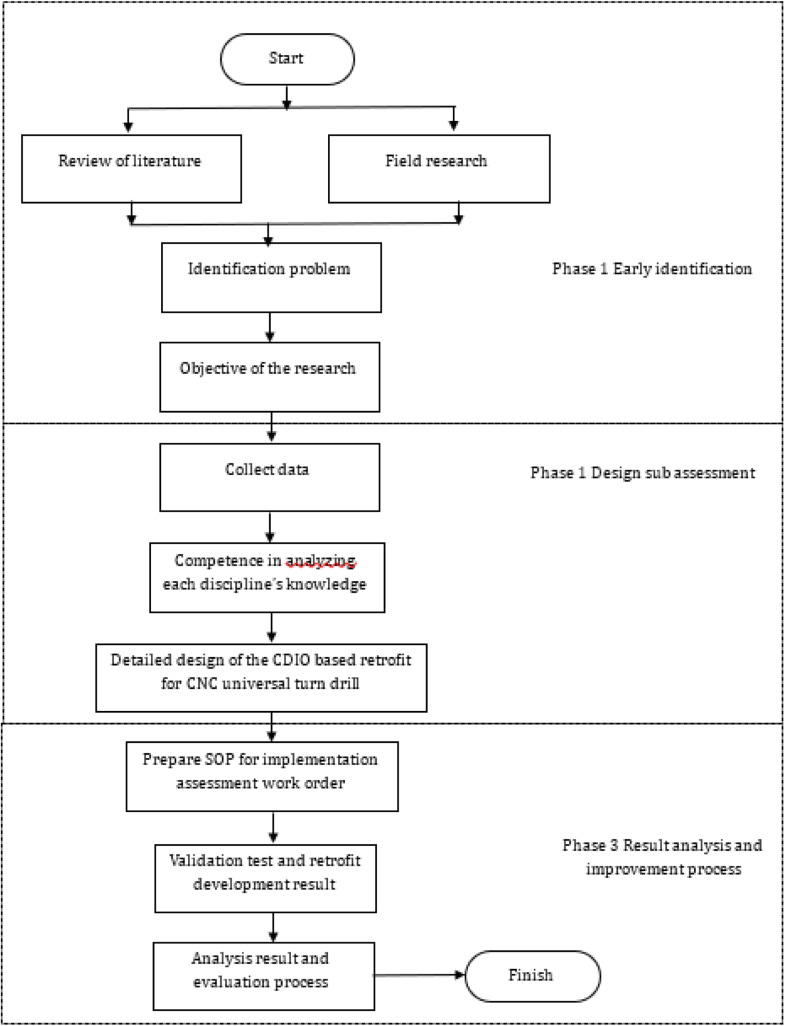
Flow diagram for resolving the CNC universal turn drill retrofit (Bachtiar & others, 2018).

The application of CDIO standards for retrofitting computer numerical control (CNC) universal turn drill is significant, as this machine represents a core competency for mechanical engineering students specializing in manufacturing and is extensively utilized in vocational education [2],[11]. This retrofit is utilized to enhance student competence in implementing programming methods and transferring coding results from computer-generated from technical drawing of workpieces to machines, enabling automatic operation in accordance with the compiled coding design. This machine is capable of processing surface work on cylindrical, conical, and flat objects, and it can also perform drilling and enlarging of holes in cylindrical or square workpieces. The availability of CNC lathe retrofits can facilitate the advancements associated with the fourth industrial revolution [4] (Herwan, et al., 2019). This CNC lathe retrofit demonstrates superior accuracy compared to conventional lathes and minimizes the need for extensive human expertise in operation, as the programming code is executed automatically by the machine. Additionally, the procurement or manufacture of CNC lathe retrofits is influenced by the high cost of new CNC lathes, which is inversely related to the relatively economical cost of retrofits. Furthermore, the retrofit work can be performed on-site, eliminating the need for relocation. The retrofit implementation into CDIO is executed sequentially, as illustrated in Fig. 4.

## Method details

In summary, Table 7 lists the specifics of the tasks and equipment needed to carry out the universal turn drill machine retrofit for a complete semester.

**Table 7:** Project activities.

No	Activities	Time to work						Tool Requirements	PIC
		Aug.	Sep.	Oct.	Nov.	Des.	Jan.		
1	Calculation of the stepper motor's needed torque and the ballscrew's minimum diameter							Laptop Manual book Universal turn drill machine Vernier caliper	All team student and leader
2	Stepper motors are integrated into the x and z axes of the model design for a retrofit universal turn drill.							Laptop and software design	All team student and leader
3	Commissioning calculation of motor torque, stepper motor type selection, minimum diameter requirements, and other technical issues							Laptop and software design	Lecture (manager project), Instructor and All team student
4	Fabrication and manufacturing of design results							Univ.turn drill Welding machine Measuring tools	All team student and Instructor
5	Product prototype testing with a Mach 3 controller							Retrofit univ. turn drill Controller mach 3	All team student
6	Validation test and evaluation of universal turn drill retrofit activity results							Instrument and rubric assessment Measuring tools	All team student, Lecture (manager project), Stakeholder and Faculty team
7	Questionnaire for consumer satisfaction								All student team

The application and implementation of CDIO highlight the necessity for students to engage actively in project activities that involve cross-disciplinary collaboration. In a group or team, a leader is responsible for assigning tasks to all members for participation in project work. The CNC lathe retrofit project involves 4–6 students from different departments, supervised by one lecturer serving as the project manager and an instructor acting as the project facilitator. The implementation stages of CDIO in this project activity require student groups to compile, design, implement, and operate the outcomes of the project activities, as illustrated in the process flow in Fig. 4. Additionally, the project manager and facilitator conduct screening and a thorough review to assess the project from both technical and non-technical viewpoints. Upon completion of the project activities, the student group submits a report detailing the project implementation design, logbook, and final results. This documentation undergoes a comprehensive review by the project manager, faculty team, and interdisciplinary expert committee to assess the validation outcomes of the CDIO, which will be incorporated into the relevant course assessment. This review of evaluation and validation pertains to instruments developed in relation to the graduate outcomes specific to each discipline within the activity group. The evaluation targets project users, specifically lecturers, students, and vocational school students, including those from vocational high schools (SMK) and polytechnics are totaling 92 respondents who have completed CNC courses.

## Calculation of minimum diameter and torque calculations in the x and z axes

**Fig. 5:**
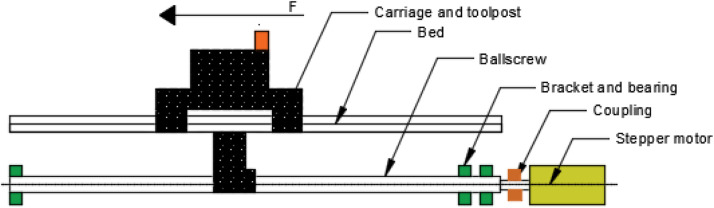
Schematic of the mass proped by the ballscrew.

(1), (2) are used to establish the minimum diameter and torque of the stepper motor to be utilized on the universal turn drill lathe on the x and z axes. Fig. 5 shows the component locations supported by the ballscrew.

(1) $$d\;\geq \;\sqrt{\frac{4F.\text{sf}}{\pi \sigma_{\text{yp}}}}$$

(2) $$T=\frac{F.d_{m}}{2}\left(\frac{l+\pi .d_{m}}{\pi .d_{m}-f.l}\right)$$

With d is diameter minimum ballscrew (mm), F: maximum force (N), sf_:_ safety factor (2.5), $\sigma_{\text{yp}}$: allowable stress of material (215 N/mm^2^). Whereas T is torque (Nm), $d_{m}$: average diameter of the minimum diameter ballscrew (mm), f: coefficient of friction (0,1–0,2), l: lead (mm).

In the meantime, Table 8 displays the mass measurement findings for each component, including those supporting the x and z axes. This data can be used to calculate the necessary stepper motor torque and the minimum diameter of the ballscrew.

**Table 8:** Mass supported by ballscrew.

No.	Item	Quantity	Mass on axis (g)	
			X	Z
1	Stepper motor	1	1700	1700
2	Ballscrew	1	500	2000
3	Coupling	1	40	40
4	Bracket	1	1000	400
5	Bearing	2	100	100
6	Bolt	6	20	20
7	Carriage components	all item	26.480	44.980

## Stages of developing the model

**Fig. 6:**
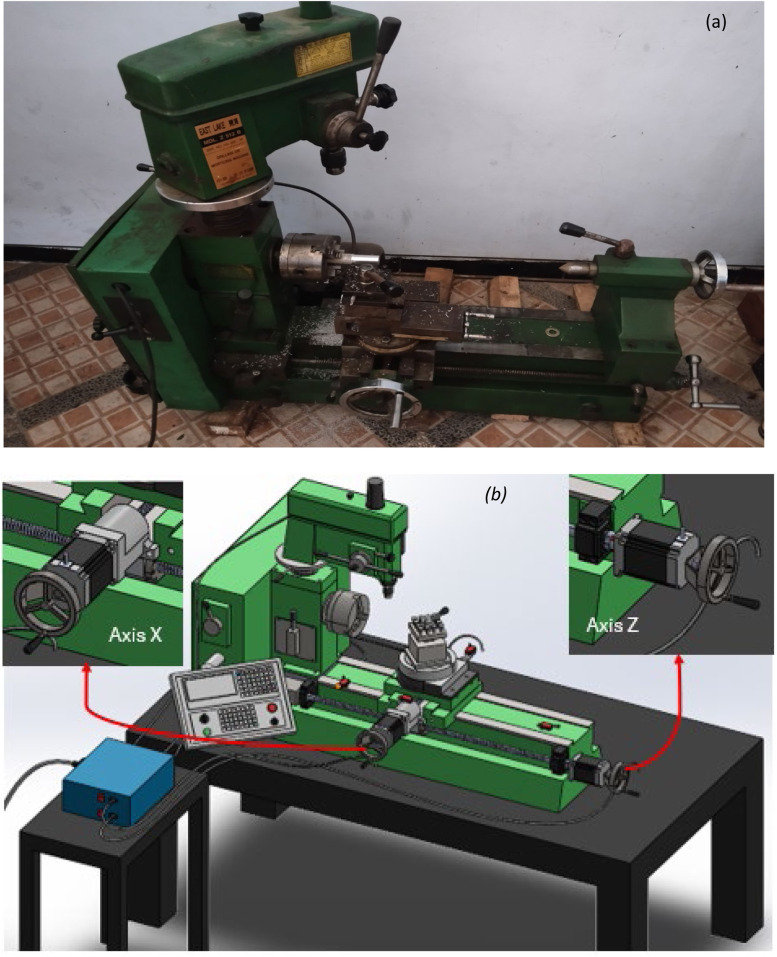
(a) Manual universal turn drill; (b) Design of the retrofited CNC lathe.

At this stage, the planning and measurement of the original conventional lathe were conducted, followed by modeling in three-dimensional form using CAD software. This stage also delineates the calculation of the requirements for the components to be replaced, taking into account functional aspects and the availability of these components in the field. Fig. 6 presents the details of the 3D modeling design.

## Developing of electrical component system

**Fig. 7:**
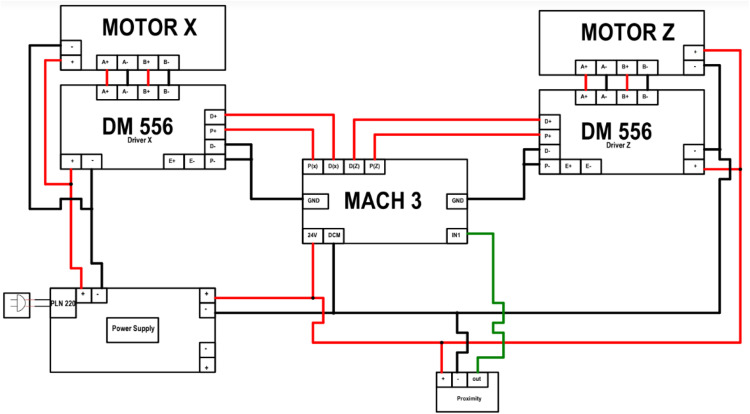
Wiring diagram for retrofitting the universal turn drill.

The electrical circuit system in the development of this CNC lathe machine involves the interconnection of various components during installation. The detailed circuit in Fig. 7 allows for an explanation of each component utilized, including the following:

a) Microdriver module DM 556
b) Stepper motor x and z axis (nema 23 with torque 2 N and 3.2 N)
c) Power supply 24 V 20A
d) Proximity DC 24V
e) Controller Mach3
f) Box and switchs

To operate the machine and achieve accurate product results, it is essential to configure the parameters of the input, output, and tuning motor in accordance with the capabilities of the stepper motor. The configuration process involves the parameterization of the tuning motor and the setup of input and output.

## Installing and configuring the prototype retrofit universal turn drill to mach 3 controller parameters

Replace Mach 3 display

**Fig. 8:**
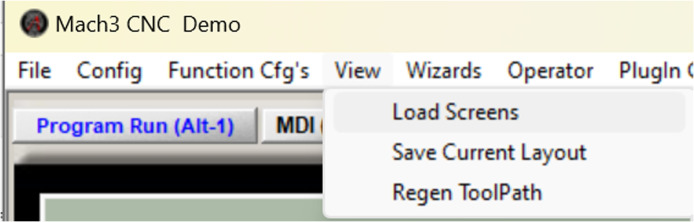
Select the load screen.

**Fig. 9:**
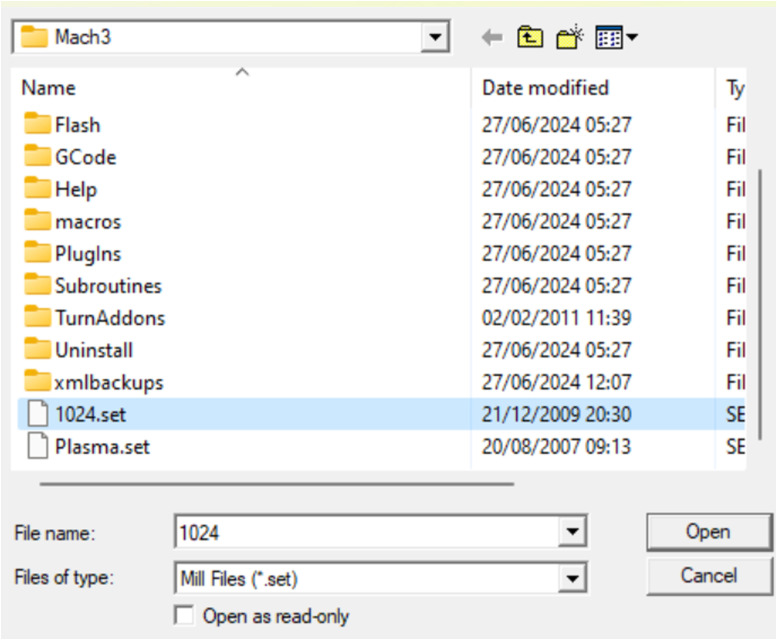
Select the name file.set.

**Fig. 10:**
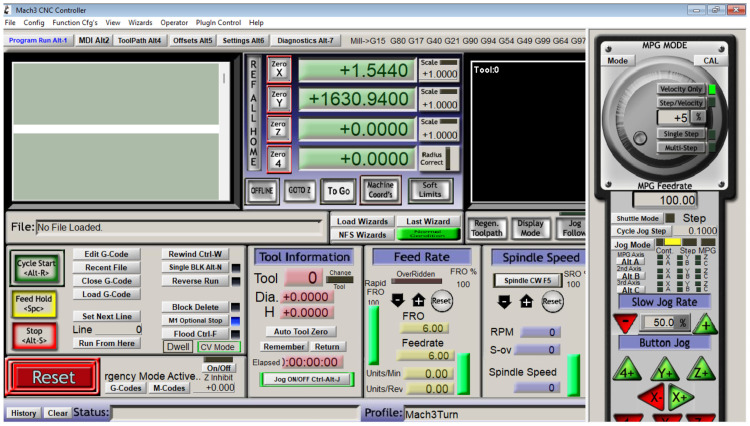
The final display of the change result through the load screen.

It is possible to change the appearance of the template application to a view that makes it easier for users to read during operation, presented in Fig. 8, Fig. 9, Fig. 10 with the steps being as follows:

Select part of view → load screens

Select the one with extension.”set” → select the file → open

Thus, the application's final look is as follows.

Stages of configuring parameterization in Mach 3

**Fig. 11:**
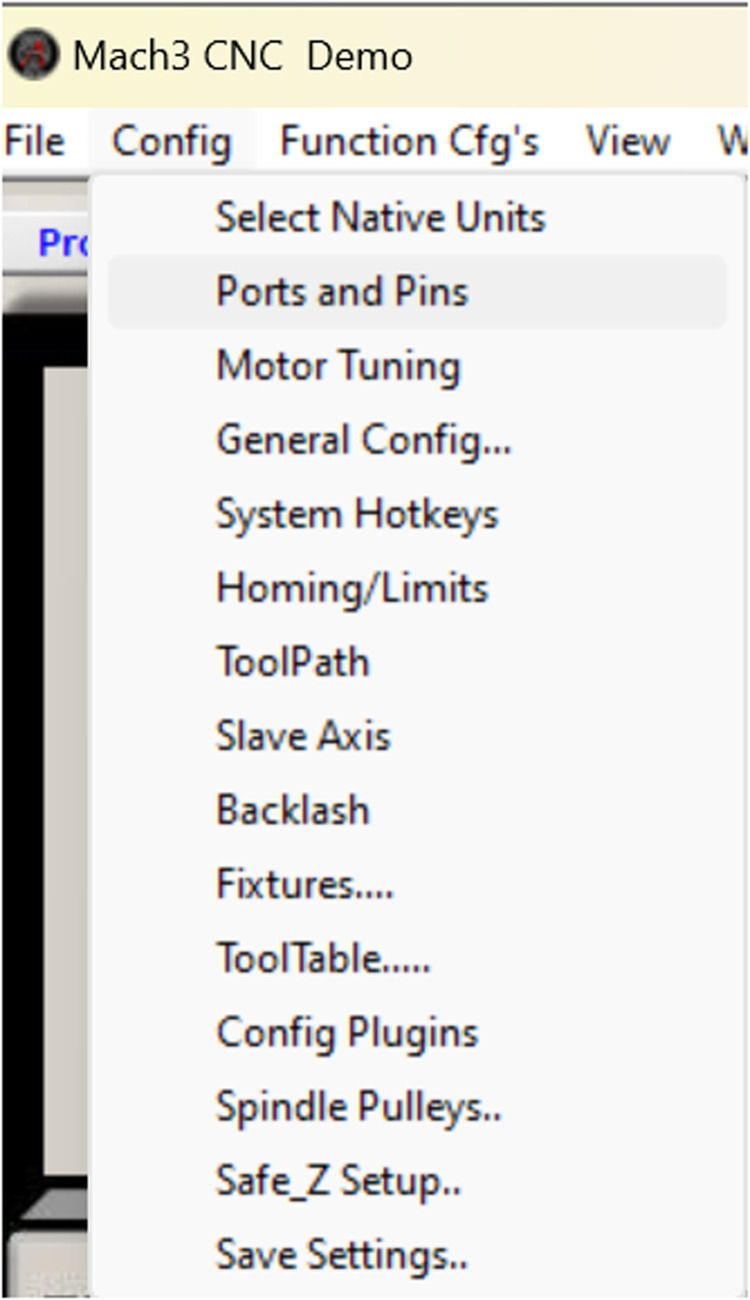
Port and pin configuration.

**Fig. 12:**
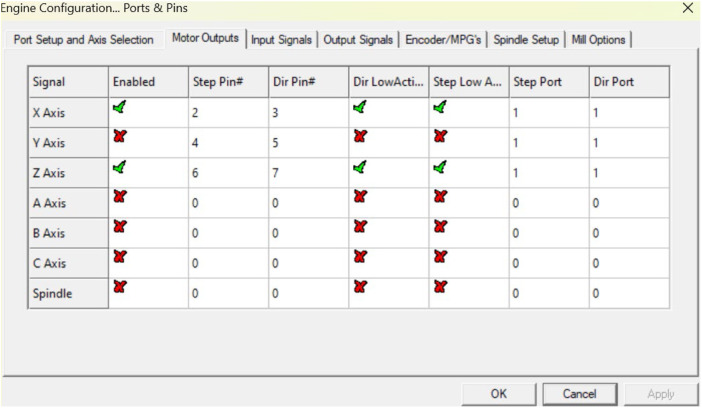
Configuration of signal, step port and dir port on output motor.

**Fig. 13:**
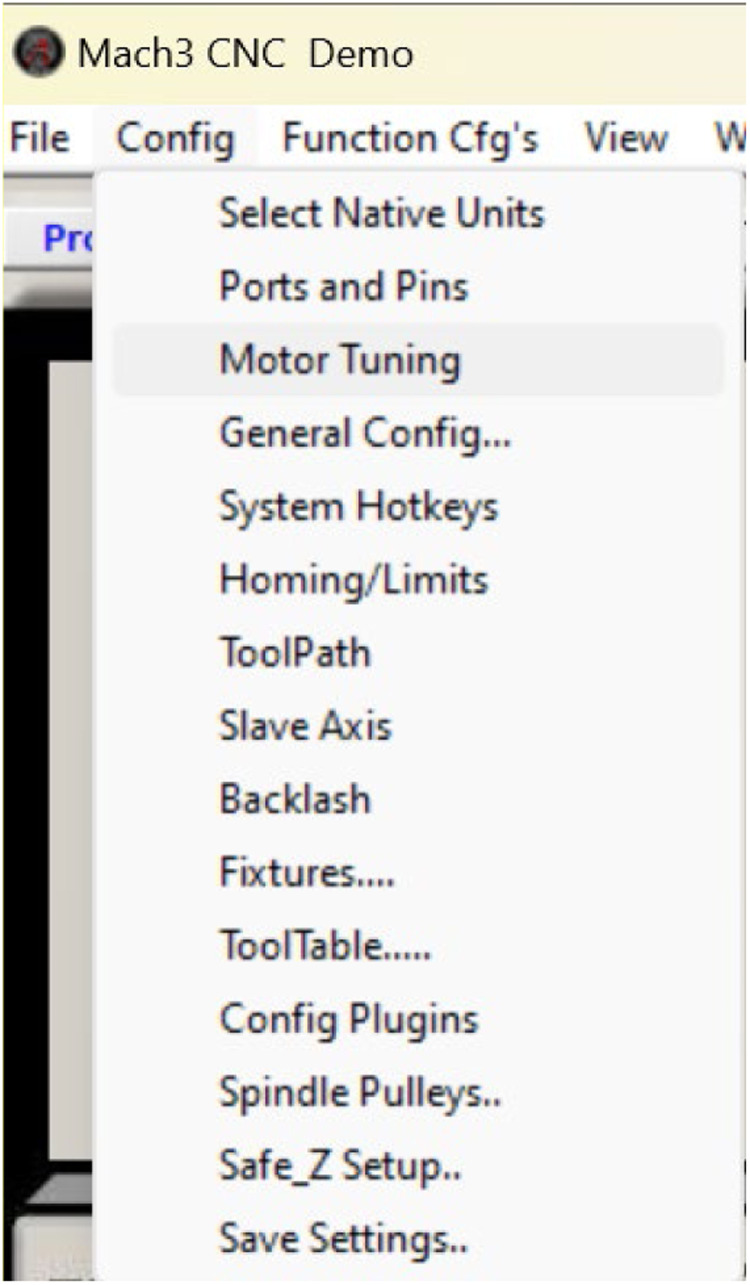
Configuration motor tuning.

Configuring the input, output, and motor tuning parameters according to the capabilities of the stepper motor is essential for correct machine operation and product output. The procedures involved in this process presented in Fig. 11, Fig. 12, Fig. 13 are as follows:

Stages in input and output configuration

Select the menu config → select ports and pins check or activate the signal on the x and z axis then change the value on the step port and dir port to 1, as attached.

Stages in tuning motor parameterization configuration

Select config → motor tuning

**Fig. 14:**
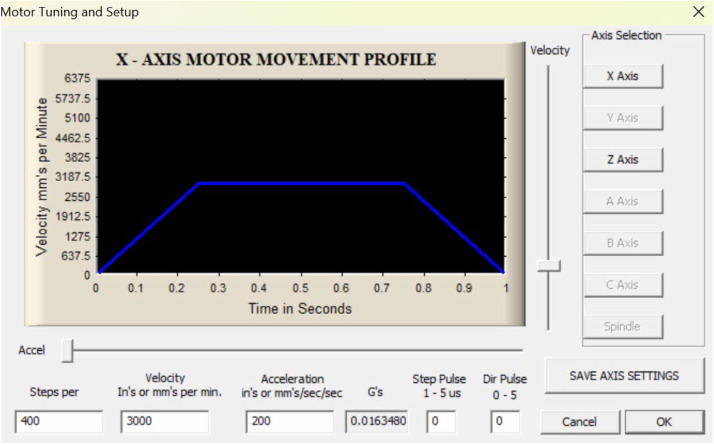
Steps per on the initial X-axis.

Select the x and z axes, then recalculate until a value is near the ballscrew size used by modifying the actual conditions of the ballscrew size used in each axis and entering the value in the step per unit based on the computation results. Each ballscrew used for the x and z axes is SFU 1204 and SFU 1605, as shown in Fig. 14.

## Implementation of machine development result

**Fig. 15:**
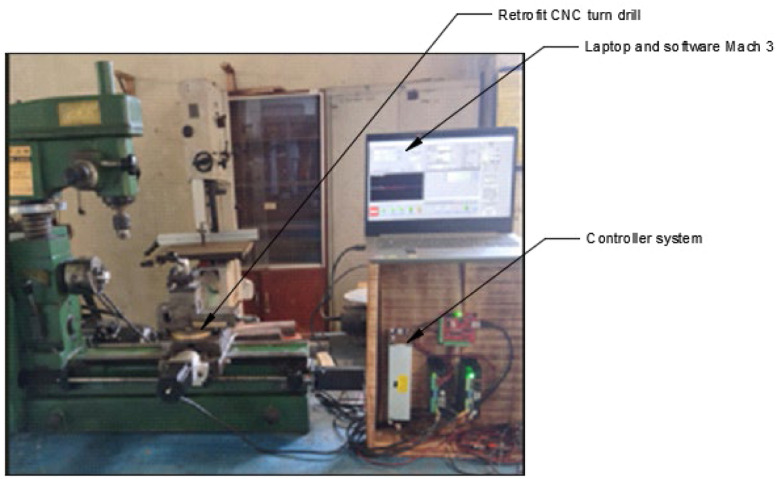
Development results of CNC lathe retrofit prototype.

Following the installation of components and electrical systems, as well as parameterization, the final step involves testing the machine and measuring the results of the workpiece using the retrofitted machine, as illustrated in Fig. 15.

## Evaluation of user satisfaction

**Fig. 16:**
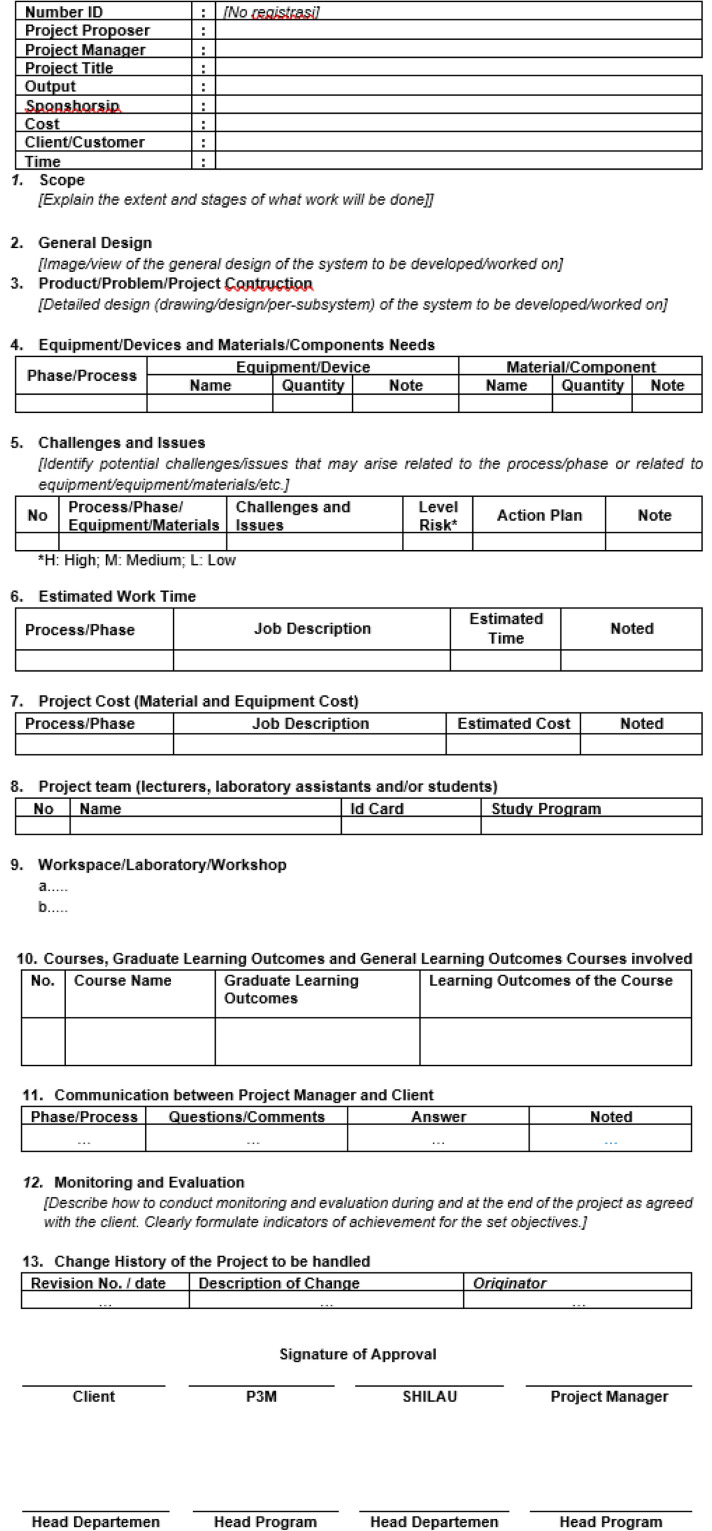
The project form for implementation design [3].

**Fig. 17:**
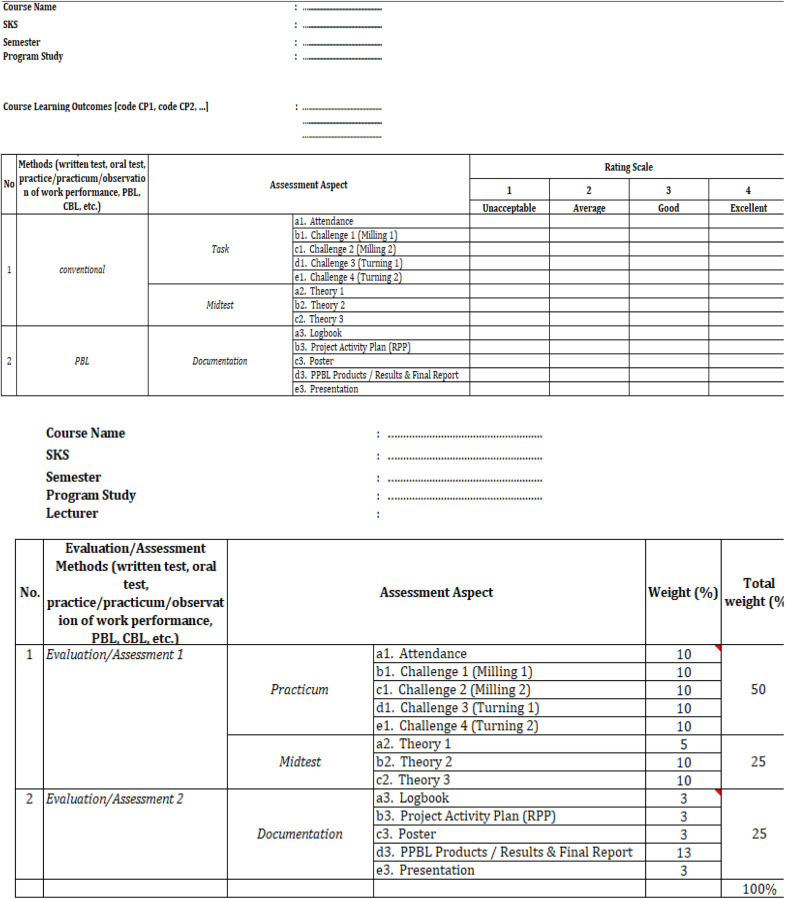
Final assessment rubric for CNC course [3].

This assessment utilized two processes, the first involving instruments from institutional quality assurance, as illustrated in Fig. 16, Fig. 17. The second approach involves the use of a follow-up questionnaire to assess the effectiveness of the CDIO method in improving the achievement of objectives related to the development of conventional CNC-based lathes, as illustrated in Table 9, Table 10. (Bachtiar & others, 2018) (Jalil, Razali, Rahman, Rahim, & Abd Samad, 2024).

**Table 9:** Answer scoring.

Answer Scale	Point
Strongly disagree	1
Disagree	2
Agree	4
Strongly agree	5

**Table 10:** Research results of the development of CNC universal turn drill.

No	Description	Strongly disagree	Disagree	Agree	Strongly agree
1	The clarity of the operating instructions for this CNC Lathe retrofit is under examination.	–	1	78	13
2	Does transitioning from a manual to an automatic sled enhance the ease of machine operation	–	1	62	29
3	Is the mach3 controller interface attractive	–	6	75	11
4	Is this cnc lathe retrofit relevant for use in vocational schools, such as: SMK and Polytechnic	–	1	55	36
5	Is the design sequence of this CNC lathe retrofit easy to understand?	–	6	73	13
6	Is the sequence of component installation steps easy to understand?	–	8	71	13
7	Whether this CNC Lathe retrofit can be used as learning media in vocational schools	–	1	59	32
8	Are the names of the main components used in this CNC Lathe retrofit easy to find in the field?	–	6	72	14
9	Is this lathe CNC machine retrofit linear with the industrial era 4.0	–	3	73	16
10	Wheter this CNC lathe retrofit needed by micro, small and medium industries	–	7	65	20

## Method validation

In the preliminary phase of planning for the implementation of activities in the CDIO framework for retrofitting CNC lathes, the student team will develop a project implementation plan. The preparation of this lesson plan requires adherence to at least 13 essential points for completeness. The team leader must allocate activities based on the competencies of the student group to ensure that they are executed effectively, coherently, and within the established time frame. Upon completion of the lesson plan preparation, the student team will engage in a discussion to validate the design with the project manager. This ensures that each parameter compiled according to the lesson plan aligns with the requirements and capabilities of the manufacturing process using existing equipment or machinery. The details of the lesson plan process sequence are illustrated in Fig. 16.

Thus, the final validation evaluation involves all student teams, project managers, stakeholders, and relevant lecturers for a thorough rubric assessment on each course actively involved in the project activities. As a case example in this project evaluation, one of the actively involved courses is Computer Numerical Control (CNC) with a fully integrated assessment rubric based on CDIO standards and syllabus as presented in Fig. 17.

## Discussion

The application involves socializing the retrofit to users, specifically students in vocational schools (SMK), mechanical engineering/mechatronics students who have completed CNC courses, and lecturers specializing in CNC Table 10 presents the results of the questionnaire indicates three primary elements:

1. Impressive is the presence of retrofit CNC lathes.

• The responses categorized as agree and strongly agree, as presented in Table 11, can be elucidated as follows:

**Table 11:** Institution-based user satisfaction level.

	Institution		
	Vocational education	Polytechnic	Lecturer
Satisfaction level			
Agree ( %)	72	78	75
Strongly agree ( %)	22	20	21
Disagree ( %)	6	2	4
Strongly disagree ( %)	0	0	0
• Operating instructions are comprehensible, with 78 respondents agreeing and 13 strongly agreeing.
• Transitioning from manual to automatic sled replacement is straightforward, with 62 participants agreeing and 29 strongly agreeing.
• Alignment with the Industry 4.0 era: 73 respondents agree, and 16 respondents strongly agree.
• 65 respondents agree, and 20 strongly agree, regarding the suitability for MSMEs.
• The findings in point (a) indicate that users have positively received the concept and implementation of CNC lathe retrofit.
• Relevance to Vocational Education:
• Points 4 and 7 indicate the following:
• This retrofit is pertinent for application in vocational schools (55 agree, 36 strongly agree).
• It can serve as a medium for learning, with 59 respondents agreeing and 32 strongly agreeing.
• The CNC retrofit demonstrates potential to enhance technology and skills-based education within the manufacturing sector.
• Challenges in Interface and Component Installation:
• While there was general consensus, notable observations emerged regarding specific aspects:
• Six respondents disagreed with the Mach3 control interface.
• Eight respondents disagreed on the clarity of the component installation sequence.
• This indicates that although the overall system received positive feedback, there are areas for enhancement, particularly regarding the user interface and installation guide.

The CNC lathe retrofit is beneficial and pertinent for vocational education and MSMEs, aligning with Industry 4.0 trends. It is essential to focus on the interface and installation aspects to enhance user satisfaction.

• Elevated satisfaction levels across all institutions:
• The proportion of respondents indicating agreement or strong agreement is notably high across all three institutions:
• Seventy-two percent agree, and twenty-two percent strongly agree, resulting in a total of ninety-four percent.
• Polytechnic: 78 % agree, 20 % strongly agree, resulting in a total of 98 % agreement.
• Seventy-five percent of lecturers agree, while twenty-one percent strongly agree, resulting in a total agreement of ninety-six percent.
• The data indicates that the majority of participants across all institutions expressed satisfaction with the assessed aspects, with the Polytechnic achieving the highest satisfaction rate at 98 %.
• The level of dissatisfaction is minimal.
• A limited number of respondents indicated disagreement, and no respondents expressed strong disagreement:
• High vocational school (SMK): 6 % expressed disagreement, while 0 % indicated strong disagreement.
• Polytecnic: 2 % expressed disagreement, while 0 % indicated strong
• disagreement
• Lecturer: 4 % express disagreement, while 0 % indicate strong disagreement.
• This suggests that there is minimal objection or dissatisfaction regarding this program or product.

3. Consistency of perceptions among institutions:

• Despite minor percentage variations, the satisfaction levels across SMK, Polytechnics, and lecturers exhibit consistency, with a majority of respondents expressing agreement or strong agreement.
• This suggests that the assessed program or product is positively received across various educational institutions, encompassing vocational students, polytechnic students, and lecturers.

Table 11 indicates a high level of satisfaction with the evaluated program or product across all institutions, with minimal instances of dissatisfaction. This demonstrates the effective implementation of CDIO that aligns with the expectations of users across various educational institutions. The final evaluation of the results of the implementation of the CNC lathe retrofit was carried out using instruments that have been standardized by Polibatam.

A comprehensive evaluation is carried out in one CNC course, with details of the evaluation instrument assessment values given in Table 12, with six criteria given. Starting from how the prototype of the universal turn drill product is produced both from the hardware components and the controller software that had been installed. Second, product demonstration, how team members was able to explain the concept and trial operation of the product in detail. Third, whether the shape of the prototype is in accordance with the initial design of the stacking and design team. Fourth, whether the development of this prototype meets the specifications of the design. Fifth, how is the contribution of each team, whether the collaboration between cross-departments is very impactful. Lastly or sixth, how the team can and is able to explain in detail in the form of a presentation and report in the form of a full and detailed report. The assessment of each individual evaluation, as illustrated in Fig. 17, is categorized into two groups: conventional and PBL. These assessments aim to evaluate and enhance students' soft skills relating to the underlying theory required for the execution of the assigned project, while also offering a detailed overview of each student's assessment. After establishing the grading criteria for each student participating in the project, the evaluation proceeds according to the grading weights assigned to each category. These weights are combined to obtain a final grade for each student activity.

**Table 12:** Criteria assessment through scoring.

Criteria	Excellent (4 Points)	Good (3 Points)	Average (2 Points)	Unacceptable (1 Point)
Prototype/model development	Prototypes or models are optimally developed, meet set specifications, and reflect innovation and creativity.	The prototype was effectively developed; however, it exhibited minor flaws and a deficiency in innovation.	The prototype was completed; however, it exhibited numerous shortcomings and failed to fully satisfy the established specifications.	The prototype failed to satisfy the anticipated criteria regarding both completeness and standards.
Project demonstration/ testing	The demonstration is conducted efficiently and effectively, showcasing all key functions as anticipated.	The demonstration was effectively conducted; however, certain deficiencies were noted in the presentation or testing components.	Demonstrations are performed; however, they lack structure, and testing is not entirely successful.	The demonstration or test was ineffective or inadequately performed.
Accomplishment of objectives	All project objectives have been successfully met with satisfactory outcomes.	The majority of objectives have been successfully achieved. The team operates effectively; however, there exists a minor discrepancy in the contributions of its members.	Certain objectives were met; however, the outcomes were insufficient. Team collaboration was suboptimal, with substantial contributions limited to a small number of members.	No goals are achieved, or the results are far below the set standards.
Team work and contribution	Teams achieve optimal performance by establishing a clear division of tasks, ensuring balanced contributions, and facilitating efficient collaboration.	Execute tasks efficiently Efficient collaboration among team members.	Perform work independently without coordination.	Teamwork is lacking, and the contributions of members are minimal.
Presentation and Report writing	Presentations are conducted with clarity and professionalism, whereas reports are systematically organized, adhere to a specified format, and are devoid of errors.	The presentation is clear, yet contains minor flaws; the report is satisfactory, but requires improvement.	The presentations and reports lacked proper structure and exhibited several deficiencies.	Presentations and reports that are insufficient, lack organization, or fail to adhere to the specified format.

## Conclusion

The implementation of CDIO (Conceive, Design, Implement, Operate) is essential in vocational faculty, vocational high schools (SMK), and polytechnics, particularly for CNC-based universal turn drill retrofit manufacturing projects, as this methodology promotes practice-oriented learning, industry relevance, and aligns with a 12-standard framework. According to the Likert scale assessment, the majority of respondents from these institutions had a high degree of satisfaction, suggesting that CDIO is beneficial in enhancing conceptual comprehension, technical proficiency, teamwork, and problem-solving capabilities. Through CDIO, students not only grasp theoretical concepts but also create prototypes that align with the requirements of Industry 4.0, such as CNC retrofits, which may be incorporated into the curriculum to enhance practical learning and address the demands of MSMEs. The efficacy of the CDIO methodology is evaluated based on multiple criteria, including enhancements in student competency, active engagement in the project, and the ultimate results of the produced items. The use of CDIO in CNC retrofit manufacturing demonstrates that the average approval percentage score above 70 %, indicating substantial success in fulfilling the objectives of CDIO implementation. Consequently, CDIO enhances students' technical competencies while equipping them for authentic industrial issues through methodical, quantifiable, and tangible outcomes.

## Limitations

Preelimery research, CDIO applied to the CNC turn drill retrofit project in the form of a product prototype still emphasizes research aspects ranging from project selection, preparation and cross-disciplinary collaboration, material and material selection process, implementation and manufacturing process, process evaluation and distribution of satisfaction to users with the controller system used based on open source with only one type of controller system, Mach 3. In the next research will be conducted using several other types of open source controller systems, so that it can be analyzed not only limited to user satisfaction but more to other spatial effects, such as changes in component size, coordinate alignment, vibration stability, backlash compensation, costs required between each type of controller and ease of maintenance.

## CRediT author statement

In this study, the authors contributed to the initial idea, hypothesis formulation, research design, as well as the drafting and editing of the manuscript. Meanwhile, supervision, including contributions to the revision and validation of the manuscript, was carried out by the authors' advisors, Prof. Suhardjono and M. Khoirul Effendi, Ph.D

## Declaration of competing interest

The authors declare that they have no known competing financial interests or personal relationships that could have appeared to influence the work reported in this paper.

## Data Availability

Data will be made available on request.

## Appendix Supplementary materials

Image, application 1

Supplementary material and/or additional information [OPTIONAL] optional Image, application 2
